# Ionizing Radiation May Induce Tumors Partly Through the Alteration or Regulation of Mismatch Repair Genes

**DOI:** 10.3390/cancers17040564

**Published:** 2025-02-07

**Authors:** Mingzhu Sun, Kevin Monahan, Jayne Moquet, Stephen Barnard

**Affiliations:** 1 UK Health Security Agency (UKHSA), Cytogenetics Group, Radiation Effects Department, Radiation, Chemical, Climate and Environmental Hazards Directorate, Chilton, Didcot OX11 0RQ, UK; 2 Lynch Syndrome Clinic, Centre for Familial Intestinal Cancer, St Mark’s Hospital, London North West University Healthcare NHS Trust, Watford Road, Harrow HA1 3UJ, UK; 3 Department of Surgery and Cancer, Imperial College London, South Kensington Campus, London SW7 2AZ, UK

**Keywords:** ionizing radiation, radiation effects, mismatch repair (MMR), Lynch syndrome, primary/secondary tumorigenesis

## Abstract

Ionizing radiation is mutagenic and carcinogenic, and it is reported to induce primary and secondary tumors with intestinal tumors being one of the most commonly observed. However, the pathological and molecular mechanism(s) underlying the radiation-associated tumorigenesis remain unclear. A link between radiation and somatic tumorigenesis partly through genetic, epigenetic alteration and/or regulation of mismatch repair (MMR) genes has been hypothesized for the first time within this review. Clinical observations and experimental findings provide significant support for this association including MMR mutations as well as altered MMR RNA and protein expressions that occurred post-exposure, although existing evidence in published literature is sparse in this niche area. Some speculative mechanisms are suggested with this review to inform future research. Further studies are needed to understand the roles of the MMR system in response to radiation and to test this possible connection which could potentially provide useful and urgently needed information for clinical guidance.

## Simple Summary

Ionizing radiation, such as X-rays used during radiotherapy treatment, can kill cancer cells, but it can also cause various types of cancers depending on the treatment site that is exposed; however, many underlying mechanisms remain unclear. Here, we propose a hypothesis based on published evidence that one of the DNA repair pathways, mismatch repair, may be involved in the normal cells turning cancerous. This pathway has not been fully investigated and its roles may be underestimated in response to radiation exposure. As part of our research efforts towards characterizing radiation effects in Lynch syndrome patients, this review can inform future studies and potentially contribute to improved treatment and long-term care and management of these patients.

## 1. Introduction

Lynch syndrome (LS) is an autosomal dominant cancer predisposition syndrome. It is the most common cause of hereditary colorectal cancer (CRC) and is also associated with an increased lifetime risk of developing other predominantly epithelial cancers, in particular endometrial, ovarian, urothelial and upper gastrointestinal cancers [1]. LS is characterized by the presence of a pathogenic germline variant in one or more of the DNA mismatch repair (MMR) genes, chiefly *MLH1*, *MSH2*, *MSH6*, and *PMS2* [2], or the deletion of the *EPCAM* (epithelial cell adhesion molecule) gene that regulates *MSH2* expression [3]. The main known function of the MMR system is to recognize and repair erroneous insertion, deletion and incorporation of bases during DNA replication and recombination [4]. A defective MMR system can lead to accelerated accumulation of somatic mutations resulting in carcinogenesis with approximately 70–90% of LS attributable to deleterious mutations in *MLH1* and *MSH2*, and 10–30% distributed between lower penetrance *MSH6* and *PMS2* genes [1,5,6]. Cancer risks can also vary between and within families due to broader genomic and gene–environment interactions [5]. A high level of somatic instability in mono- or di-nucleotide tandem repeats is another characteristic feature of MMR-deficient tumors, termed microsatellite instability (MSI) [7]. Almost all LS-associated tumors contain insertion and/or deletion of these short repetitive DNA sequences [8,9]. The frameshift mutations of microsatellite repeats within coding genes are believed to be the mechanism that drives carcinogenesis [10]. LS affects approximately 1 in 400 individuals; however, only 5% of patients with LS are aware of the diagnosis in the UK [1].

Ionizing radiation (IR), such as X- and γ-rays used during radiotherapy treatment, can cause a wide range of direct and indirect DNA damage depending on the dose, dose-rate and technical settings used, such as base damage, single strand breaks, double strand breaks, abasic sites, DNA–protein crosslinks, inter- and intra-strand crosslinks, etc., as well as free radical-containing reactive molecules generated through radiolysis of water [11,12]. There are close connections between radiation-induced DNA damage in cells and the onset of mutations in genes through DNA mis-repair and the subsequent development of cancer [13]. Radiation-induced damage to the DNA coding for MMR proteins should be the same as that to other genes and is considered as a stochastic effect. Significantly, younger age is associated with a greater susceptibility to radio-carcinogenesis due to a higher proportion of actively proliferating cells, which are more sensitive to radiation-induced damage [14]. In the adult human body, the intestinal epithelium has the highest proliferation rate with normal complete turnover every 3–5 days [15], and endometrium in adult women also retains regenerative capacity attributable to the stem cells residing in the basalis layer of the tissue [16]. This may explain why such tissues with actively dividing epithelial cells in LS patients could be more prone to tumorigenesis following DNA damage and mis-repair.

In response to radiation, the MMR system is involved in IR-induced DNA damage recognition and signaling, G2/M phase cell cycle arrest, nucleotide excision, repair of DNA and clustered DNA damage, cytotoxicity, and apoptosis [17,18,19,20,21,22]. Additionally, MMR deficiency is linked to increased tumor mutational burden by allowing the accumulation of mis-repaired DNA damage [6], and MMR proteins are also associated with homologous recombination suppression [23]. Many other protein components participating in various DNA metabolic pathways are essential for mismatch repair [22]. Consequently, defects in MMR are associated with genome-wide instability, predisposition to cancer, resistance to chemotherapeutic agents, abnormalities in meiosis and sterility in mammalian systems [22,24]. Furthermore, germline mutation carriers in other genes, e.g., *POLE* and *MUTYH*, can develop CRC with MMR deficiency [25]. These findings suggest that the roles of MMR proteins are not limited to mismatch repair and their functions in response to radiation could have been hugely underestimated hitherto.

Mismatch repair has been extensively studied. A schematic diagram of the MMR pathway and the role of each MMR protein were presented in detail by Tamura et al. [26] and reviewed by many others previously [17,22,24,27]. Briefly, on the identification of newly synthesized DNA, mismatch recognition is mediated by the formation of MutS heterodimers: MutSα (MSH2 and MSH6) for single base-pair mismatches and small insertion–deletion loops, or MutSβ (MSH2 and MSH3) for larger insertion–deletion loops. MutSα binds around double-stand DNA and interacts with MutL, another heterodimer comprising MLH1 with either PMS2 or PMS1, to form a tetrameric complex. Together with many other components recruited to the site, mismatched base or single strand will be removed and replaced. Despite the well-studied roles of MMR proteins, how each of them reacts to radiation and how they coordinate in different molecular pathways with each other and with other cellular components following radiation exposure are still unknown.

**Figure 1:**
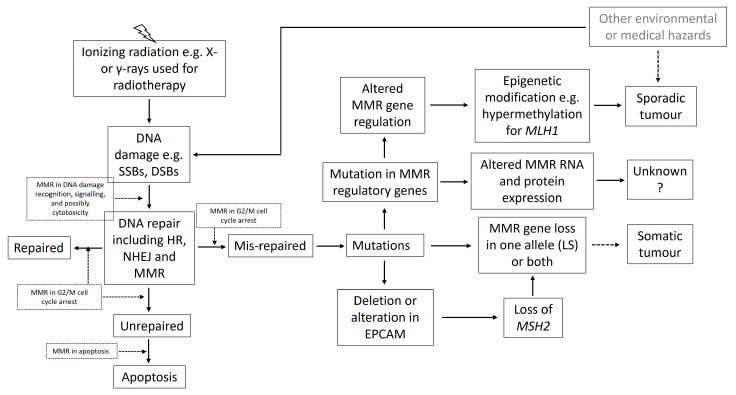
Ionizing radiation (IR) can directly or indirectly cause various types of DNA damage, which can be repaired, mis-repaired or unrepaired depending on the severity of the damage. MMR proteins participate in the recognition of the damage, the cell cycle arrest at G2/M phase to allow DNA repair, and the removal of mis-incorporated bases and erroneous insertions and deletions, together with two of the major DNA repair systems—homologous recombination (HR) and non-homologous end joining (NHEJ). MMR proteins may also be involved in cell cytotoxicity and apoptosis. Mis-repaired DNA can occur in MMR genes affecting either one allele or both. For LS patients, damage to the normal allele can trigger tumorigenesis. For cells with no germline deficiency, the loss of function in both alleles is required, which can be induced by IR according to the “two-hit” hypothesis. DNA damage that occurs in MMR regulatory genes can result in alteration in the MMR gene (e.g., epigenetic hypermethylation in *MLH1* linked to sporadic tumors) and the expression of RNA and protein, although the consequence is unknown. There are many other internally or externally generated factors (such as environmental or medical hazards shown in grey text) that may cause MMR gene mutation or modification leading to dysfunction; therefore, tumors can occur independent of exposure to radiation. The roles of MMR in dashed boxes are still under study as well as the pathways indicated by dashed arrows.

An illustrative figure is presented alongside this review to assist with the understanding of the possible involvement of MMR in response to radiation exposure (Figure 1). Briefly, IR as well as many other internally or externally generated mutagens can damage the genetic material resulting in mutations or cell death. Cells have developed complex mechanisms to repair DNA damage including homologous recombination (HR), non-homologous end joining (NHEJ), base excision repair (BER), nucleotide excision repair (NER) and mismatch repair (MMR) [28]. Each of these pathways plays a crucial role in maintaining genomic stability. DNA damage, mis-repair and subsequent mutation can occur in MMR genes affecting either one allele or both. With the presence of inherited germline defect(s) in MMR genes, LS patients have an increased risk of losing function in the MMR system and developing cancer after radiotherapy. In comparison, people with no germline deficiency have much reduced risk. When MMR regulatory genes are damaged by IR, epigenetic modification to *MLH1* can happen leading to the development of sporadic cancer. Compromised MMR regulatory mechanism may also alter the expression of MMR RNAs and proteins even though the consequence is currently unknown.

The molecular mechanisms underlying the development of CRCs following exposure to radiation are unclear. LS is recessive at the cellular level and the development of somatic tumors in LS patients essentially would require the loss of function in both alleles of an MMR gene [5]. This requirement could be met by either the presence of a germline variant or a primary hit to one allele, plus a secondary hit to the other allele in separate events during radiotherapy when repeated irradiation at high doses is inflicted at the same treatment site. Supporting evidence for this “two-hit” hypothesis can be found in MMR-deficient sporadic tumors with no germline mutations or promotor methylation where two somatic mutations were acquired in separate events [25,29]. This review amalgamates some of the existing evidence that supports the association between radiation exposure and MMR alteration in terms of possible IR-induced genetic and/or epigenetic mutation and IR-stimulated modification in RNA and protein expression from clinical observations to experimental studies.

## 2. Association Between Radiation and MMR Gene Mutations

There appears to be a clear association between somatic MMR gene mutation and previous anti-cancer treatment.

### 2.1. Hodgkin’s Lymphoma

Rigter et al. [30] reported that Hodgkin’s lymphoma survivors treated with infra-diaphragmatic radiotherapy or procarbazine-containing chemotherapy have a 5-fold increased risk of developing CRC. Therapy-related CRCs (t-CRCs) had a more frequent loss of MSH2/MSH6 staining (13% vs. 1%, *p* < 0.001) when compared with CRCs in the general population, even though no difference was found in the loss of MLH1/PMS2 staining and *MLH1* promoter methylation between these two groups. For MSI-positive CRCs without *MLH1* promoter methylation, double somatic MMR gene mutations or loss of heterozygosity were detected in 7/10 (70%) of t-CRCs and 8/36 (22%) of CRCs in the general population (*p* = 0.008). Importantly, MMR gene mutations in t-CRCs were classified as pathogenic and these t-CRC cases could not be ascribed to LS.

In a more recent study, Rigter et al. [31] reported an increased risk of developing gastric cancer in patients treated for Hodgkin lymphoma or testicular cancer with radiotherapy. Molecular subtyping of 90 gastric tumor tissues from these patients post-radiotherapy revealed that in 6 MSI-positive samples (6.7%), 3 had *MLH1* promoter methylation, 2 had double somatic mutations in MMR genes, and 1 had a homozygous deletion involving *MLH1* gene that was found in the tumor sample, but not in normal tissue. There was also a MSI stable sample that showed partial loss of MLH1 and PMS2 staining.

### 2.2. Prostatic Adenocarcinoma

Albeit a small sample size, in 1 of 21 cases (4.8%) of prostatic adenocarcinoma deep deletion of *MSH2* and *MSH6* was reported in patients who received prior radiotherapy for prostate cancer. This deletion spanning both the *MSH6* gene and part of the adjacent *MSH2* gene resulted in microsatellite instability and a very high mutational burden [32].

### 2.3. Case Studies

A 19-year-old male developed an anaplastic astrocytoma (AA) within the irradiation field 7 years after radiotherapy (total dose: 56.6 Gy) treatment for sellar/suprasellar craniopharyngioma. Deeper screening performed on tumor-derived genomic DNA revealed isocitrate dehydrogenase 1 mutation (*IDH1*-R132H) and two concurrent mutations in this radiation-induced AA: a missense mutation for *TP53* and a short in-frame deletion for *MLH1*. It was speculated that the *MLH1* alteration may have been induced completely by radiotherapy or could have been triggered by the combination of *IDH1* and *TP53* mutations.

A 40-year-old male with a history of leiomyosarcoma of the thigh and male breast cancer diagnosis at age 36 was treated with a compartmental resection followed by post-operative radiotherapy and a right mastectomy, respectively. He then developed two secondary tumors—colon cancer at 48 years and prostate cancer at 50 years. Immunohistochemical (IHC) staining revealed the loss of MLH1 staining in the breast carcinoma and sarcoma, and a high level of MSI was detected in the sarcoma. Molecular analysis identified a missense variant in the *MLH1* gene in the family [33]. The authors speculated that the patient’s later cancers could be radiation induced because scatter dose was used and secondary tumors may occur both within and outside the radiation field [33]. This case shows that radiation could have caused the loss of function in the normal allele of the *MLH1* gene during the post-operative radiotherapy, which led to the development of tumors at other sites, although IR-induced alteration in other unidentified genes is also possible. Similarly, a post-radiation sarcoma was found in a 74-year-old man with known Muir–Torre syndrome and confirmed *MSH2* germline mutation, who developed pleomorphic liposarcoma in a previous radiation field and IHC revealed loss of expression of MSH2 and MSH6 [34].

Two patients with a family history of colorectal carcinoma developed rectal carcinoma 17 and 26 years post pelvic radiotherapy for carcinoma of the uterine cervix. Mutation in exon 4 of the *MLH1* gene was detected in one of the patients. No mutation in *MLH1* or *MSH2* was revealed for the other patient. In both cases, the tumors arose in a previously irradiated area and were histologically distinct from the primary tumors. Radiation-associated rectal carcinoma is rare, but may occur in patients with a family history of colorectal carcinoma including LS [35].

In the case of a 45-year-old woman diagnosed with endometrial cancer (EC), IHC showed the absence of MSH2 and MSH6 protein expression in the pelvic mass tissue 5 years after receiving radiotherapy (^125^I radioactive seeds implantation) followed by six cycles of chemotherapy. Deletions of exon 8 and 9 in *EPCAM* and deletions of exon 1 and 8 in *MSH2* were revealed by genetic testing using peripheral blood [36]. The existence of germline mutations in *EPCAM* and *MSH2* in combination with the radiation-induced mutation to the normal allele of one or both of these genes could be the cause of MSH2 and MSH6 loss and the secondary cancer in the pelvis. MSH6 only binds with MSH2 and the loss of MSH2 automatically leads to the loss of MSH6 staining (lynchscreening.net).

Relevant to these clinical findings, radiation exposure has been reported to accelerate the intestinal tumor growth in *Mlh1*-knockout mice [37] and to increase the number of tumors in *Mlh1*^−/−^ mice with dextran sodium sulphate-induced mild inflammatory colitis [38]. Thus, it is possible that radiation may not only be associated with MMR mutation, but it may also be involved in the subsequent acceleration of the growth and spread of tumors with MMR deficiency, although further evidence is needed.

## 3. Association Between Radiation and MMR Gene Regulation

In addition to the stimulation of MMR gene alteration, radiation may also regulate MMR genes, which can be shown directly by the expression of MMR RNA and indirectly by the expression of MMR proteins as well as the change in MSI post-exposure.

### 3.1. MMR Gene Expression

Quantitative real-time PCR (qPCR) was performed to analyze the MMR gene expression levels for workers (*n* = 30) occupationally exposed to IR with non-exposed workers (*n* = 25) as control. Total RNA from both groups was extracted from peripheral blood. Compared to the control group, a 5-fold increase (*p* = 0.006) was observed in the *MSH2* gene expression for those exposed to radiation as well as a weak but significant correlation (*p* = 0.041) with the *MSH6* gene, particularly when the duration of exposure was associated, and the longer the exposure time, the greater the expression of this gene [39].

Blood samples from the inhabitants in a high radiation background area (*n* = 30) and a normal radiation background area (*n* = 30) were analyzed and compared for the expression of *MLH1* and *MSH2* genes by qPCR. A significant up-regulation of *MLH1* in residents of the high background radiation area (*p* < 0.0001) was observed regardless of sex. However, when age is considered in comparison to the matched controls, there was a significantly increased expression of *MLH1* in the group of people aged above 50 years, but not in those aged below. No significant difference was found in the expression of the *MSH2* gene between residents living in different radiation background areas. Nevertheless, the expression of this gene was affected by both sex and age [40].

In one animal study, female Apc^Min/+^ mice (6–8 weeks old; *n* = 20) with C57BL/6J background received a whole-body exposure to 1.88 Gy of proton radiation either in a single acute exposure or a fractionated exposure delivered across four days. Significantly higher intestinal tumor number and grade were observed in the acute irradiated group compared to the sham and fractionated exposure groups. Immunoblot analysis showed down-regulation of MSH2 protein, and real-time PCR showed decreased expression of *Msh2* and *Mlh3* genes as well as unchanged *Mlh1* and *Pms2* genes following the acute exposures. In contrast, elevated expression of these genes was observed after fractionated irradiation in comparison to the control group [41]. This study indicates that the alteration in the expression of MMR genes may be dependent on the radiation dose, dose-rate and the setting of the exposure (acute, fractionated, or chronic) and possibly the type of radiation used in the study as well.

### 3.2. MMR Protein Expression

Over 450 germline variants have been described for MMR genes in humans (https://www.insight-group.org, accessed on 2 June 2022), with the majority of these alterations recognized as pathogenic due to the expression of truncated proteins. MMR protein expression may be used as indirect evidence to show the effect of IR on MMR gene expression even though protein expression levels cannot be predicted by transcript levels in many scenarios [42]. For example, IHC staining for MLH1, MSH2, MSH6 and PMS2 proteins was performed for 20 patients (mean age: 61.2 +/− 11.2 y) with stage II–III rectal cancer. Paraffin-embedded materials from both colonoscopy before neoadjuvant radiotherapy and the operation post-treatment were analyzed. It was found that the mean score of the stain was significantly higher for all four MMR proteins (*p* < 0.001) in the pre-treatment material as compared to the post-treatment material—MLH1 (11.2 ± 2.5 vs. 5.3 ± 3.9), MSH2 (11.0 ± 2.0 vs. 6.0 ± 3.7), MSH6 (10.8 ± 2.3 vs. 4.3 ± 2.4) and PMS2 (8.4 ± 4.4 vs. 3.5 ± 3.5). Additionally, null or weak stain was observed in 5.0% (4/80) of the pre-treatment samples as compared to 23.8% (19/80) of the post-treatment material [43].

### 3.3. Change in Microsatellite Instability (MSI)

As a characteristic feature of LS, MSI may be used as a surrogate marker for MMR deficiency [7]. It should be noted that MSI is found in 10–40% of sporadic colon, endometrial, ovarian and other cancers, and the loss of MMR function in sporadic tumors was primarily caused by hypermethylation of the *MLH1* promoter leading to its reduced expression [8]. Therefore, MSI does not necessarily indicate MMR deficiency or mutation. Notwithstanding, the changes in MSI have been associated with previous exposure to radiation and may provide supporting information for further investigation of MMR function. For example, the occurrence of MSI was less commonly seen in spontaneous tumors (16%) than in X-ray (23%) and neutron-induced tumors (83%) [7]. When compared with CRCs in the general population, Hodgkin’s lymphoma survivors treated with radiotherapy or chemotherapy had a higher MSI frequency (24% vs. 11%, *p* = 0.003) and more frequent loss of MSH2/MSH6 staining (13% vs. 1%, *p* < 0.001). The higher frequency of MSI among these therapy-related CRCs was postulated to have resulted from somatic MMR gene mutations [30].

## 4. Possible Mechanisms

The mechanistic understanding as to how radiation could lead to carcinogenesis through MMR gene mutation or regulation remains to be fully characterized. Some speculative possibilities are presented in this section to inform future investigations.

Firstly, IR is one of the known inducers of epigenetic modification [44]. MMR deficiency may lead to epigenetic changes in the DNA packaging protein histone H3 methylation profiles prior to tumor development as observed in a mouse model. Herberg et al. [45] demonstrated that the intestinal tissue of 4-month-old *Msh*2*^−/−^* mice exhibited genome-wide epigenetic changes compared to control mice with matching age, even though intestinal tumors were not observed in these mice until the age of 12 months. These authors also reported that when comparing sham-irradiated with X-irradiated *Msh*2*^+/+^* mice at 0.5 Gy, radiation-associated differences in the histone H3 methylation profiles were very similar to those induced by *Msh*2 loss. Similarly, changes in H3K36me3, an epigenetic modification to histone H3, were reported to be clearly present four weeks after irradiation or after *Msh*2 loss [45]. It should be noted that these findings were only related to *Msh*2 in mice. There is insufficient evidence to demonstrate the involvement of other MMR genes and what their roles could be in the epigenetic regulation of histone and/or chromosomal DNA.

Secondly, spontaneous mutation rate for *Msh*2-knockout male mice (*Msh*2^−/−^) at the endogenous expanded simple tandem repeat (ESTR) DNA loci was significantly higher than that in isogenic wild-type (*Msh*2^+/+^) and heterozygous (*Msh*2^+/−^) mice, whereas the spontaneous ESTR mutation rate in the heterozygous *Msh*2^+/−^ males was similar to that in the wild-type *Msh*2^+/+^ control. Additionally, a significant increase in ESTR mutation was observed in the irradiated *Msh*2^+/+^ and *Msh*2^+/−^ mice, with no detectable increase in the mutation rate found in the irradiated *Msh*2^−/−^ mice [46]. Given the heterozygous status of LS patients in the MMR genes, radiation may also induce a mutator phenotype in ESTR and other genes, such as oncogenes, tumor suppressor genes, growth control or survival genes, and subsequently accelerate the radiation-induced tumorigenesis.

Furthermore, microRNAs (miRNAs, 21–23 nucleotide-long noncoding RNAs [47]) play important roles in tumorigenesis of various cancers and radiotherapy can increase the expression of miR-155-5p and miR-760 in non-small cell lung cancer cells [48]. Overexpression of miR-155 has been reported to significantly down-regulate the core MMR proteins (MSH2, MSH6 and MLH1) and to induce a mutator phenotype and MSI; and an inverse correlation between the expression of miR-155 and the expression of MLH1 or MSH2 proteins was found in human CRC [49]. Several MSI tumors with an unknown cause of MMR inactivation also showed miR-155 overexpression [49]. In addition, miR-21 expression was also increased with radiation dose in all three human esophageal squamous cell carcinoma cell lines (i.e., TE-1, EC1 and KYSE140) after γ-radiation at doses of 2, 4, 6 and 8 Gy in comparison to unirradiated cell; and miR-21 that targets MSH2 and MSH6 mRNA has been found to be overexpressed in MSI-high CRC [50]. It is therefore possible that radiation directly or indirectly causes an increase in the expression of certain types of microRNAs, which subsequently suppress the expression of MMR proteins. IR has also been reported to affect several microRNAs (e.g., miR-29, miR-141 and miR-152) that specifically target DNA methyltransferases and may lead to subsequent alterations in DNA methylation [44].

## 5. Discussion

In this review, a possible novel association has been identified between previous exposure to IR and subsequent somatic MMR gene alteration and/or regulation that may lead to tumorigenesis through increased mutation rate, epigenetic modification and/or microRNA regulation. Germline mutation can also happen if the reproductive organs are exposed to IR. It could affect future generations, but it is beyond the scope of this review. The evidence for this potential link is clear, although based on limited published studies thus far. The lack of more supporting cases is presumably caused by the years and decades of follow-up required for the related tumors to manifest and be reported [51]. After such a long delay, it can be challenging to establish a connection retrospectively, particularly when the currently used modern screening techniques were not available at the diagnosis of the primary tumors many years prior. In addition, CRCs are often complicated with other synchronous or metachronous tumors in many cases [33]; thus, the underlying causative factors are difficult to identify. Furthermore, radiotherapy is frequently combined with other treatments, particularly chemotherapy; therefore, primary and/or secondary tumorigenesis may not be attributable to radiation alone.

Histopathological and molecular characteristics of treatment-associated CRC in Hodgkin’s lymphoma cases have demonstrated that these tumors are heterogeneous in terms of MSI status, CpG island methylator phenotype status and the mutation status of several oncogenes similar to those found in sporadic CRC [30]. In addition, radiation-induced MMR gene alteration can be genetic and/or epigenetic. It may therefore be necessary to monitor the gene modification in irradiated tissues at multiple sites in follow-up testing using multigene panels to include pathogenic variants in less validated MMR genes, such as MSH3 [25]. Importantly, caner occurrence is tissue specific, and the cis-regulatory cancer risk variants shared by different tissues may also need to be considered to inform secondary cancer surveillance strategies [52].

Unlike somatic tumors that require double mutations in MMR genes to develop, the existence and high prevalence of a mutation in MMR genes in LS patients impose a much higher risk for them to develop cancer when exposed to radiation or any other source of DNA-damaging agents. The significance in establishing a potential association between radiation and MMR gene alteration can help to raise awareness that radiation-induced primary and/or secondary tumorigenesis may be much higher than currently understood, especially in children and young people. NICE guidelines (DG27 and DG42) recommend universal testing of newly diagnosed colorectal and endometrial cancer for LS based on the consistent evidence in the cost-effectiveness and clinical benefit of a structured diagnostic pathway and subsequent cascade testing in families (https://www.bsg.org.uk, accessed on 2 June 2022). The proper implementation of these guidelines in clinical services would facilitate the investigation of radiation effects in this specific group of patients. Ongoing studies are being conducted using cells isolated from biopsy tissues and peripheral blood lymphocytes within UKHSA. Cytogenetic analysis, cell survival and DNA damage repair approaches will be investigated. These will provide the urgently required information for the effects of radiation on MMR. Together with the studies involving gene modification, expression and regulation such as sequencing, epigenetic and microRNA-related investigations, these would contribute to the improved treatment and long-term management of LS patients.

## 6. Conclusions

In this review, a possible association between radiation-induced MMR gene mutation and/or modification with the development of epithelial tumors is reported. Therefore, for LS patients the potential introduction of post-radiotherapy genetic and epigenetic monitoring of the irradiated tissues at multiple sites using the multigene panel may lead to early-stage diagnosis and ultimately improved preventative interventions through targeted screening and surveillance. Comprehensive study is required to understand the roles of MMR proteins in response to radiation exposure in addition to DNA mismatch repair. Integrated research involving radiobiology, physics and clinical studies is important to reduce the risks of treatment-related second cancers. Additionally, no radiotherapy strategies may need to be investigated and prompted for the carriers of MMR pathogenic variants.

## Data Availability

Not applicable.
